# Diel Surface Temperature Range Scales with Lake Size

**DOI:** 10.1371/journal.pone.0152466

**Published:** 2016-03-29

**Authors:** R. Iestyn Woolway, Ian D. Jones, Stephen C. Maberly, Jon R. French, David M. Livingstone, Donald T. Monteith, Gavin L. Simpson, Stephen J. Thackeray, Mikkel R. Andersen, Richard W. Battarbee, Curtis L. DeGasperi, Christopher D. Evans, Elvira de Eyto, Heidrun Feuchtmayr, David P. Hamilton, Martin Kernan, Jan Krokowski, Alon Rimmer, Kevin C. Rose, James A. Rusak, David B. Ryves, Daniel R. Scott, Ewan M. Shilland, Robyn L. Smyth, Peter A. Staehr, Rhian Thomas, Susan Waldron, Gesa A. Weyhenmeyer

**Affiliations:** 1 Lake Ecosystems Group, Centre for Ecology & Hydrology, Lancaster, United Kingdom; 2 Environmental Change Research Centre, Department of Geography, University College London, London, United Kingdom; 3 Department of Meteorology, University of Reading, Reading, United Kingdom; 4 Eawag, Swiss Federal Institute of Aquatic Science and Technology, Department of Water Resources and Drinking Water, Dübendorf, Switzerland; 5 Institute of Environmental Change and Society, University of Regina, Regina, Canada; 6 Department of Biology, University of Regina, Regina, Canada; 7 Freshwater Biological Section, University of Copenhagen, Copenhagen, Denmark; 8 King County Water & Land Resources Division, Seattle, Washington, United States of America; 9 Centre for Ecology & Hydrology, Bangor, United Kingdom; 10 Marine Institute, Furnance, Newport, Co Mayo, Ireland; 11 Environmental Research Institute, University of Waikato, Hamilton, New Zealand; 12 Scottish Environment Protection Agency, ASB Eurocentral, North Lanarkshire, Scotland; 13 Kinneret Limnological Laboratory, Israel Oceanographic and Limnological Research, Migdal, Israel; 14 Rensselaer Polytechnic Institute, New York, New York, United States of America; 15 Dorset Environmental Science Centre, Ontario Ministry of the Environment and Climate Change, Dorset, Ontario, Canada; 16 Centre for Hydrological and Ecosystem Science, Department of Geography, Loughborough University, Loughborough, United Kingdom; 17 Center for Environmental Policy, Bard College, New York, New York, United States of America; 18 Department of Bioscience, Aarhus University, Roskilde, Denmark; 19 Cyfoeth Naturiol Cymru/Natural Resources Wales, Maes-y-Ffynnon, Bangor, Wales; 20 School of Geographical and Earth Science, University of Glasgow, Glasgow, Scotland; 21 Department of Ecology and Genetics/Limnology, Uppsala University, Uppsala, Sweden; University of Wisconsin Milwaukee, UNITED STATES

## Abstract

Ecological and biogeochemical processes in lakes are strongly dependent upon water temperature. Long-term surface warming of many lakes is unequivocal, but little is known about the comparative magnitude of temperature variation at diel timescales, due to a lack of appropriately resolved data. Here we quantify the pattern and magnitude of diel temperature variability of surface waters using high-frequency data from 100 lakes. We show that the near-surface diel temperature range can be substantial in summer relative to long-term change and, for lakes smaller than 3 km^2^, increases sharply and predictably with decreasing lake area. Most small lakes included in this study experience average summer diel ranges in their near-surface temperatures of between 4 and 7°C. Large diel temperature fluctuations in the majority of lakes undoubtedly influence their structure, function and role in biogeochemical cycles, but the full implications remain largely unexplored.

## Introduction

Temperature is one of the most fundamental drivers of ecosystem structure and function. It affects rates and equilibria positions of chemical reactions [1] and rates of metabolic processes, especially amongst poikilothermic aquatic organisms [2–4]. In lakes, temperature has a pervasive effect on a large range of physical, chemical and biological attributes and processes and influences the physical structure, rates of photosynthesis and respiration [5], biological growth rates [6], organic carbon mineralization [7], greenhouse gas emissions [8–10], organism size [11], the timing of phenological events [12], the likelihood of toxic cyanobacterial blooms [13], and the available habitat for fish species [14]. Quantification of surface temperature variation, and the factors that control it, are therefore of paramount importance in understanding lake behaviour and function.

Lake temperatures have been measured around the world for many years at weekly to monthly frequencies, enabling an understanding of causes and wide-ranging ecological consequences of seasonal, annual and decadal temperature changes (e.g. [15]). Knowledge of diel temperature cycles is also important for, among other things, calculating biogeochemical reaction rates and gas fluxes accurately, elucidating systematic differences between different lakes and for determining whether the predominance of day-time measurements has biased limnological understanding. Until recently, the continuous high-frequency measurements required to resolve diel cycles have not been sufficiently wide-spread to allow a systematic examination of diel temperature changes to be undertaken. However, the recent establishment of scientific networks dedicated to the collaborative analysis of high-frequency data, such as GLEON (http://www.gleon.org/) and NETLAKE (https://www.dkit.ie/netlake/), have provided an opportunity for a large-scale analysis of diel temperature variability. Thus, we have collated data from 100 lakes, that are deep enough to stratify, across four continents (S1 Table, S1 Fig), for which high-frequency temperature measurements were available, to determine the extent, and causes, of variation in diel surface temperature cycles.

Upper water temperature is controlled by heat exchange across the air-water interface, which in turn is determined predominantly by incident solar radiation, cloud cover, air temperature, relative humidity, and wind speed [16]. It is also influenced by the depth of the upper mixed layer and light attenuation in the water column (e.g. [17]). To understand the overarching controls on diel temperature cycles we have, therefore, examined the influence of four integrating variables that might be expected to have a strong effect: i) latitude, which determines variation in solar radiation flux via day-length and insolation; ii) altitude, which affects air temperature via the adiabatic lapse rate; iii) lake area, which is the primary influence on thermocline depth within a lake [18]; and iv) in-lake attenuation of photosynthetically active radiation (PAR, spectral band 400 to 700 nm), which influences the vertical distribution of incoming solar radiation within a lake and is also known to influence the vertical thermal structure [19].

## Methods

Near-surface (~1 m), in situ, water temperature measurements of high-frequency (data resolution ranged from minutely to hourly, and accuracy of between 0.001°C and 0.2°C; specific details (S2 Table) of the thermistors used in each lake are available upon request) were collated from 100 temperate and boreal lakes (totalling more than 200 thousand measurements) on four continents. No specific permissions were required for any of the lakes studied in this investigation and the data used were collected previously for other studies. The field studies did not involve endangered or protected species. The lakes varied in surface area between about 2.5 x 10^3^ m^2^ and 1.6 x 10^8^ m^2^, light attenuation of PAR (K_d_) between 0.08 m^-1^ and 5.7 m^-1^, in altitude between -211 m a.s.l. and 2464 m a.s.l., in (absolute) latitude between 32.817° and 59.846°, and in maximum depth between 2 m and 256 m. Of the 100 lakes, 74 had direct or indirect measurements of light attenuation, and 24 had both meteorological measurements (wind speed, solar radiation, air temperature and relative humidity) and temperature profiles (S1 Table). Unfortunately, the majority of the lakes included in this investigation did not have information on the temporal variations in K_d_ thus we only had single values for each lake. This may be problematic, as K_d_ varies through time with, among other things, algal production which can have a large influence on the thermal dynamics of lakes (e.g. [20–22]), but similar to other large-scale studies (e.g. [23]) we were restricted to single values in this study.

The diel temperature range (DTR) of the near surface water was calculated as the difference between the maximum and minimum daily surface temperature for each lake. For the 24 lakes with meteorological data and depth-resolved temperature measurements, an approximate theoretical DTR (ΔT_0_) was also calculated:

$$\Delta {\text{T}}_{0}\;=\frac{\left(\Delta \text{t}\cdot {\text{A}}_{0}{\cdot \text{Q}}_{\text{zmix}}\right)}{\left(\rho_{0}\cdot {\text{C}}_{\text{pw}}\cdot {\text{V}}_{\text{zmix}}\right)},$$ (1)

where Δt is the heating period for a given day (taken to be 12 h, or 43200 s), A_0_ is lake surface area (m^2^), Q_zmix_ is the net heat flux to the upper mixed layer (J m^-2^ s^-1^), ρ_0_ is the density of the surface water (kg m^-3^), C_Pw_ is the specific heat of water at constant pressure (4186 J kg^-1^°C^-1^), and V_zmix_ is the volume of water (m^3^) within the upper mixed layer (z_mix_, m). The depth of the upper mixed layer was defined as the first depth where the temperature difference was estimated to be greater than 0.2°C relative to the temperature located at 1 m (e.g. [17]). To determine the volume of water within the upper mixed layer, bathymetric maps were required. Hypsographic curves for each lake were extracted from these maps, where available, or from GPS/depth-sounder data. For locations where neither of these datasets where available, the lakes were assumed to have a conical shape constrained by surface area and maximum depth (e.g. [23]). The amount of surface heating which influences the upper mixed layer, Q_zmix_, was estimated following the methods detailed in Woolway *et al*. [17], by using the ‘Lake Heat Flux Analyzer’ program [24].

### Statistical methods

To investigate the controls of the DTR, we used a Generalised Additive Model (GAM) with a gamma error distribution and the logarithm link function. Specifically, the GAM was used to examine the relationship between the average summer DTR and the explanatory variables. Four predictor variables (available for 74 lakes) were used to explain the variation in DTR: lake surface area (A_0_), K_d_, latitude (φ), and altitude (h). Absolute latitude was used, calculated as distance from the equator i.e. irrespective of hemisphere, and a logarithmic transformation was applied to lake surface area. The light attenuation coefficient was converted to a percent transmission per metre, *I*_z_, with the following formula:

$$I_{z}=100\times \text{exp}\left(-K_{d}\right)$$ (2)

We used a GAM of the following form:

$$\mu_{i}=g{\left(\eta_{i}\right)}^{-1}\;=\;g{\left(\beta_{0}+f_{1}(A_{0i})+f_{2}(\varphi_{i})+f_{3}(h_{i})+f_{4}(I_{zi})\right)}^{-1}$$ (3)

where μ_i_ is the expectation of the response Y_*i*_ (μ_*i*_ ≡ E(Y_*i*_)) for the *i*th observation, *g* is the logarithm link function, the inverse of which maps values from the linear predictor, η_*i*_, on to the scale of the response. η_*i*_ consists of a constant term, β_0_, plus four smooth functions, *f*_*j*_, *j* = {1, 2, 3, 4}, one function per covariate considered.

To implement model selection we used a double penalty approach [25], which adds a penalty on the null space of the smoother to the usual wiggliness penalty used to select the smoothness of the *f*_*j*_. By penalizing the null space of the smoother in addition to the wiggliness penalty, individual smooths can be completely removed from the model, thus providing a principled means of model selection. Smoothness parameters and estimates of the model coefficients (i.e. β_0_ and coefficients for the *f*_*j*_) were calculated using restricted maximum likelihood [26]. Models were fitted using the R programming language [27] with the mgcv package (version 1.8–3; [28]) using the select = TRUE option for the double penalty. The method used to formulate the test statistics and *p*-values follows Wood [28] and is a test against the null hypothesis of zero effect for the *j*th smooth.

In addition to the method described above, we also followed a multi-model inference approach to determine the relative importance of each of the predictor variables in determining the DTR. The R package MuMIn [29] was used to select the best possible combination of predictor variables (= 2^N^, where *N* is the number of predictors) contained within the GAM. Models were compared using an adjusted Akaike Information Criterion (AICc). AICc is a measure of model performance, which compares the maximum-likelihood estimate of models, adjusted for increasing complexity. The model with the lowest AICc is considered to exhibit the best performance of the set tested. All models with AICc values to within four of the model with the lowest AICc value were then selected as a ‘confidence set’, thus including all possible models possessing a considerable level of empirical support. The confidence set of models was used to derive relative importance values for each explanatory variable. Relative importance, which represents the probability of a variable being present in the best-performing model for a particular predictor, was calculated in MuMIn using the relative Akaike weights of models within the confidence set.

### Ecological and biogeochemical consequences of the diel surface temperature range

We made simple calculations to illustrate the potential biases of different diel temperature cycles on estimates of temperature-dependent ecological processes. For these calculations, we assumed a mean temperature of 20°C (i.e. the mean summer temperature for the 100 lakes included in this investigation) and computed synthetic temperature cycles with amplitudes of 0.5, 3.5, and 7.5°C (equalling a diel range of 1, 7, and 15°C, respectively). These synthetic temperature cycles were computed using simple sine waves with the appropriate amplitudes (e.g. Asin(2π(1/24)t)+T_mean_; where t = 1:24, A is amplitude, and T_mean_ is the mean temperature). It is anticipated that the diel temperature cycles of lakes will not always follow a perfect sinusoid, but these temperature cycles are used for illustration here. Using these synthetic cycles we calculated the temperature-dependent CO_2_ solubility from the equation in Rebsdorf *et al*. [30] and an atmospheric CO_2_ partial pressure of 400 ppm; and oxygen solubility was calculated from equations in Mortimer [31]. Q_10_ values (i.e. the factor by which a biological rate is increased by 10°C rise in temperature) were calculated for a value of 2, typical of photosynthesis and respiration and a value of 4, typical of methane emissions from lakes. Therefore, we estimated a diel cycle in each of these temperature-dependent processes and then calculated the differences between the estimated value at 20°C (i.e. the mean temperature) and that estimated at the time of maximum temperature during the diel cycle (e.g. 23.5°C for a diel cycle with an amplitude of 3.5°C). Our objective here is to illustrate the potential bias resulting from measurements taken at different times during the day. Accounting for diel temperature variability can potentially lead to large differences in estimates of temperature-dependent ecological processes.

## Results and Discussion

**Fig 1:**
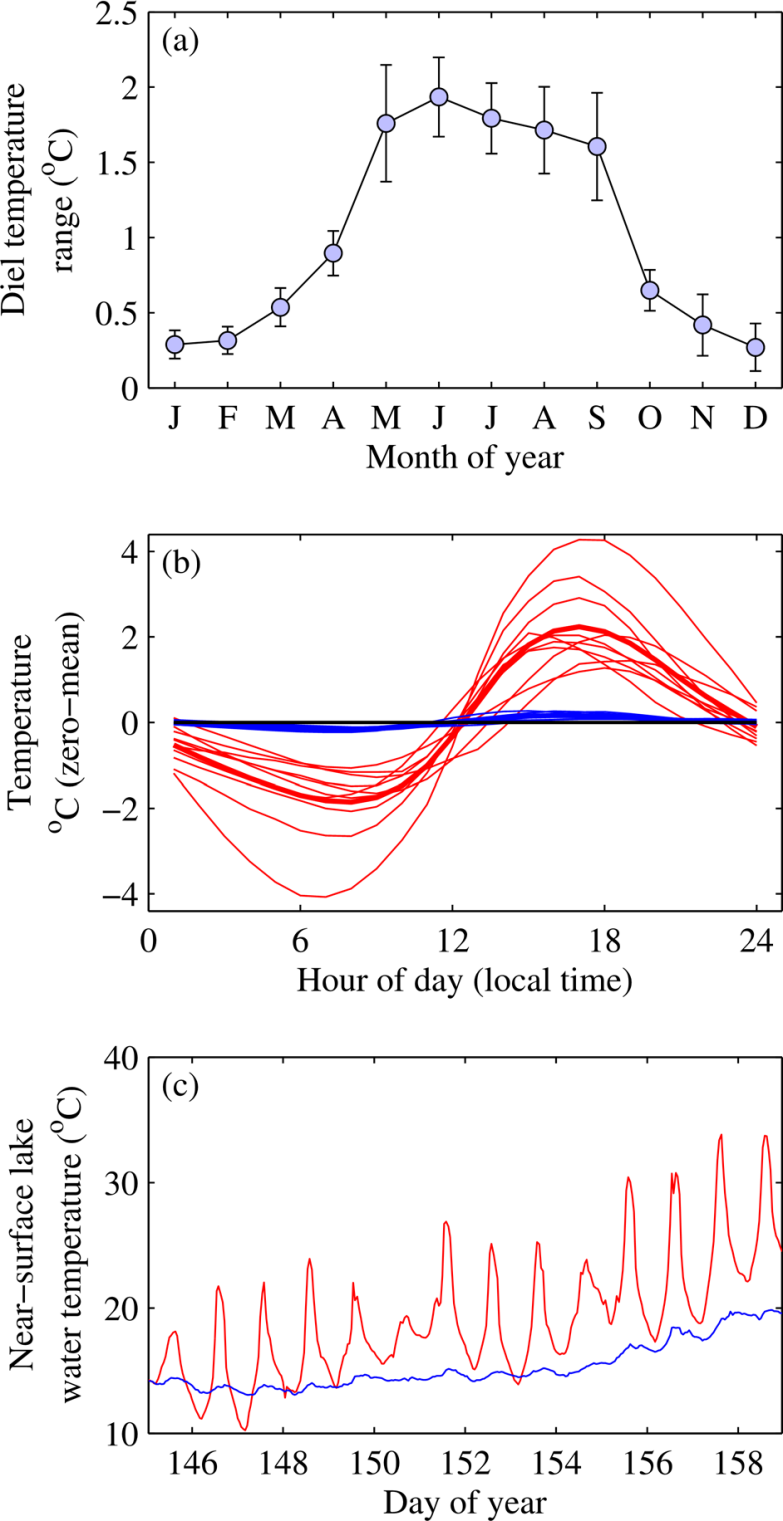
Temporal variability in near-surface lake water temperature. (a) Seasonal variability in the diel temperature range for 96 Northern Hemisphere lakes with 95% confidence intervals (note that not all lakes had data for the whole year). (b) Individually normalized (zero-mean) summer average diel cycle for the lakes that had the highest (red) and lowest (blue) 10% of diel temperature ranges measured. The bold lines represent the mean diel cycle for the 10% considered and the horizontal black line indicates zero. For clarity, we excluded Jekl Bog, which had the highest diel cycle, from this figure. (c) Example of hourly-resolution near-surface lake water temperature variation at Jekl Bog (surface area 2.5 x 10^3^ m^2^, red), and Sparkling Lake (surface area 6.2 x 10^5^ m^2^, blue), both situated in Wisconsin, USA.

The average DTR varied seasonally across the 100 lakes investigated in this study, being largest in summer and lowest in winter (Fig 1A). We subsequently restricted our analysis to summer as this is the season during which insolation and temperature normally peak along with most key biogeochemical processes. Mean summer DTR’s differed vastly (i.e. nearly 30-fold) between lakes (Fig 1B). Differences appeared unrelated to geographical proximity, as illustrated by the much greater diel cycle of Jekl Bog, Wisconsin, relative to that of neighbouring Sparkling Lake (Fig 1C). In fact, the temperature range in a single day in Jekl Bog was close to the entire annual temperature range of Sparkling Lake. The non-significant influence of geographical proximity has also been shown by Woolway *et al*. [17] for five lakes in the English Lake District and was also found for the 100 lakes studied here by investigating the relationship between the distances among lakes to differences in the diel changes in water temperature.

**Fig 2:**
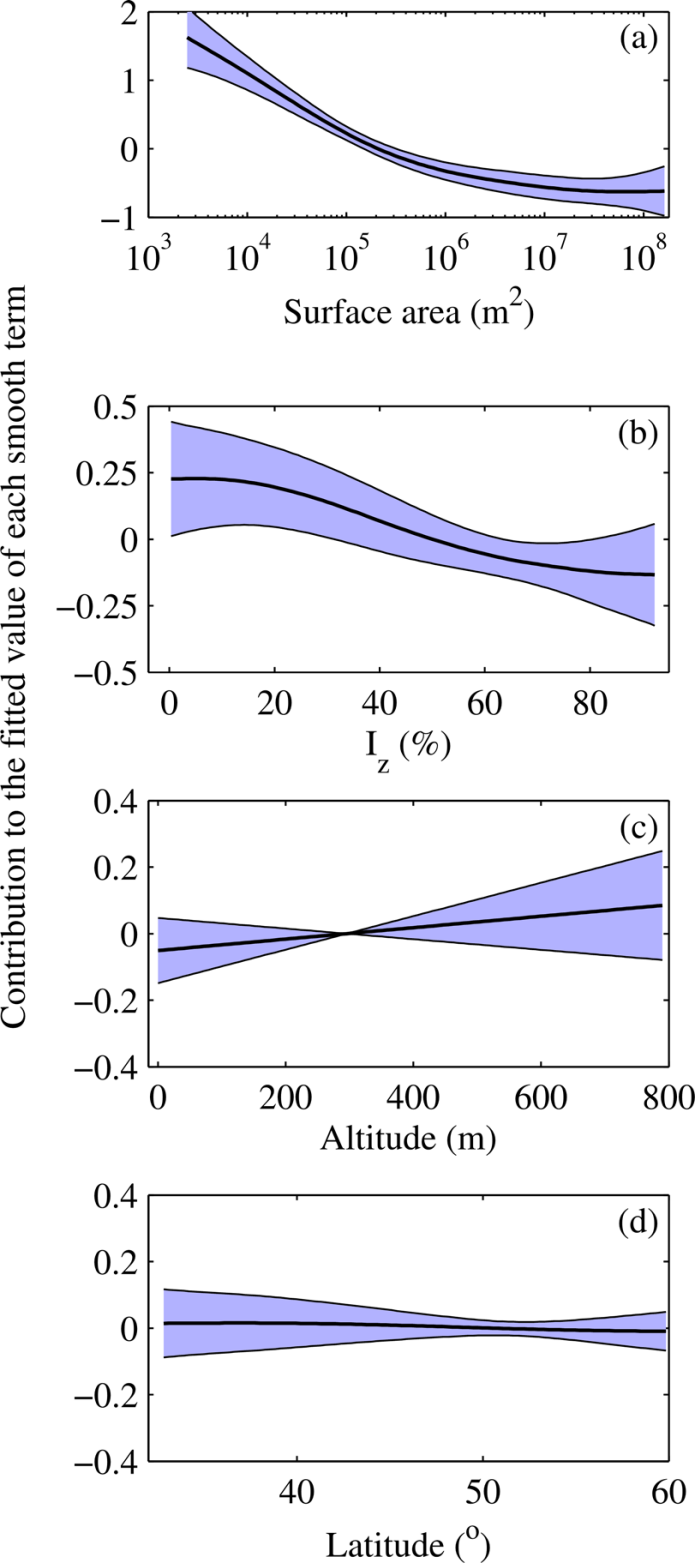
Fitted splines for the Generalised Additive Model. The y-axis is the additive contribution of the spline to the fitted model over the range of the covariate. The smooth functions are subject to centring constraints and are plotted here on different scales for clarity. The shaded region is an approximate 95% confidence interval on the function; however, it excludes uncertainty in the model's constant term, β_0_, hence the narrowness of the interval at the “middle” of the distribution for the smooths of altitude and latitude.

Summary output from the statistical model described in Eq 3 illustrates that lake area and I_z_ are identified as the most important covariates related to the mean summer DTR (Table 1). The altitude and latitude of these lakes appear to have little relationship with the DTR, especially the latter, whose contribution in the model had been shrunk considerably and is effectively zero. The fitted smooth functions illustrate how the effect on the response varies over the observed range of the covariate (Fig 2). The model suggests that the DTR is affected by I_z_ only in the least transparent waters, at values of I_z_ < 30%, but is affected by lake surface area throughout. Thus, surface area was by far the most significant explanatory variable, although I_z_ was also significant, while latitude and altitude were not significant when lake area and I_z_ were taken into account. This result was in agreement with that of the multi-model inference method (Table 2). However, calculated AICc weights indicated that, for each of the predictor variables under consideration, no single statistical model received overwhelming support for explaining the differences in the DTR among the lakes. Rather, sets of top-ranking models received similar levels of support. More importantly though, the top models consistently included a lake surface area effect (Table 2), and for the candidate set of models, lake surface area had the highest importance, followed by *I*_*z*_, latitude and altitude. This is not to say, however, that latitude and altitude would not influence the DTR if a larger distribution of lakes were examined. However, for the lakes studied here, their influence was not statistically significant.

**Table 1:** Summary output from the fitted statistical model. Summary of the model used to describe the influence of surface area (A_0_), the percent transmission per metre (I_z_), altitude above sea level (h), and latitude (φ), as shown in Eq 3, on the diel surface temperature range. EDF is the effective degrees of freedom for the spline representing each covariate. Ref. DF is the reference degrees of freedom used in the statistical test of “no effect” for each smooth. F is the test statistic and *p* the approximate *p*-value of the test. *I*_*z*_ is the percent transmission per meter.

*f*_*j*_	EDF	Ref. DF	F	*p*
A_0_	3.126	9	11.67	≪0.001
*I*_*z*_	1.565	9	0.81	0.008
h	0.529	9	0.12	0.149
φ	0.164	9	0.02	0.291

**Table 2:** Summary output from the multi-model inference approach. The relative contributions of surface area (A_0_), the percent transmission per metre (I_z_), altitude above sea level (h), and latitude (φ) are shown. Confidence set of models ranked according to their adjusted Akaike Information Criterion (AICc) statistic.

Model	A_0_	I_z_	h	φ	AIC_c_	ΔAIC_c_	Akaike weight
1	✓	✓	✓		88.0	0.00	0.278
2	✓	✓			88.2	0.13	0.260
3	✓	✓		✓	89.1	1.07	0.162
4	✓			✓	89.8	1.80	0.113
5	✓	✓	✓	✓	89.8	1.83	0.111
6	✓		✓	✓	91.9	3.88	0.040

**Fig 3:**
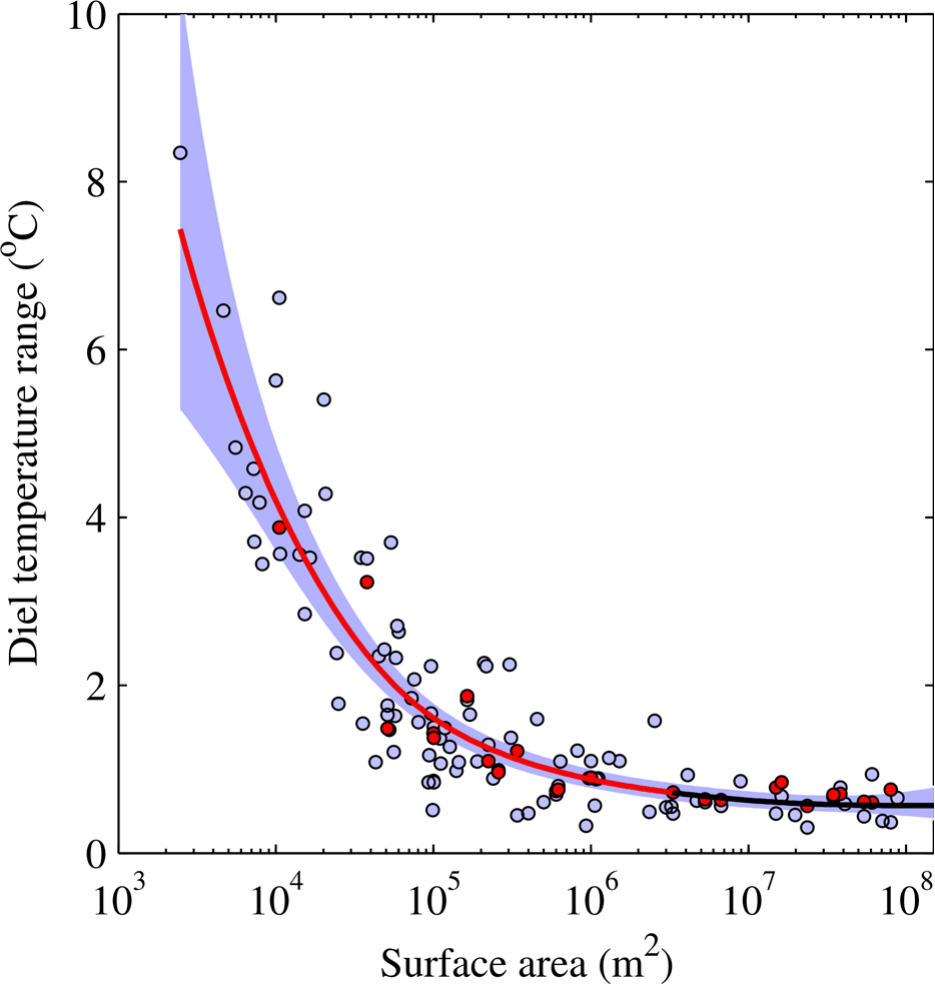
Relationship between the diel range in lake surface water temperature and surface area. Relationship between the observed (light violet circles) and theoretical (red circles) diel surface temperature range with lake area during summer, with the solid line illustrating the fitted generalised additive model with 95% confidence interval shown by the shaded region; lake surface areas where the diel temperature range changes significantly (P < 0.001) are shown with a red line.

Our analysis demonstrated clearly that for the 100 lakes analysed in this investigation, lake area is the principal determinant of the DTR. Therefore, we used a separate model to describe the relationship between the DTR and lake surface area alone. Similar to Eq 3, a GAM with a gamma error distribution was chosen to model the DTR as a function of lake surface area. For this model, 100 lakes were used. Lake area alone explained over 80% of the variation in the mean summer DTR (R^2^ = 0.83, P < 0.001, n = 100), which was only slightly lower than that explained (81.5%) by the model that also included the other predictor variables (i.e. I_z_, altitude, and latitude). The DTR was found to increase sharply with decreasing lake surface area (Fig 3). This was also shown by our theoretical approximation of the DTR (Eq 1; R^2^ = 0.88 between theoretical and observed DTR), which was calculated by utilising the further subset of 24 lakes for which continuous high-resolution meteorological and vertical temperature profile data were available, and assuming that heat absorbed within the upper mixed layer is equally distributed. The relationship between lake area and the theoretical DTR followed a pattern that was statistically indistinguishable from that calculated with observed data (RMSE = 0.6°C; Fig 3). This suggests that systematic variation of upper mixed depth with lake size is the dominant factor determining the DTR in these 100 lakes, far outweighing geographical influences on heat fluxes. This was also demonstrated by Woolway *et al*. [17] and is illustrated in Eq 1, which indicates that the DTR would be proportional to the reciprocal of the mean mixed depth. Mixed depth is known to increase with lake area [32], but diminishingly so as area increases. The results therefore indicate that, for small lakes, the change of upper mixed depth with lake area has more influence on the DTR than does the geographical variation in daily heat flux, suggesting fundamental differences in how surface heating is distributed in small lakes.

**Fig 4:**
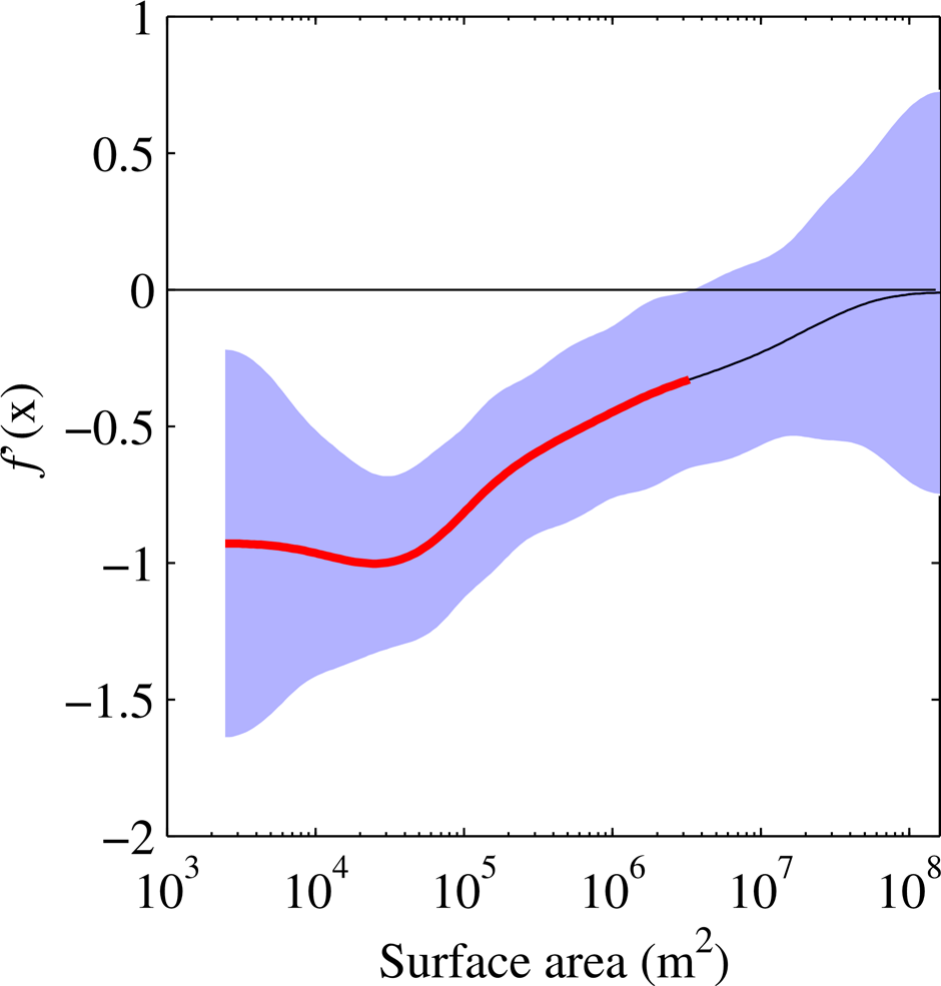
First derivatives of the fitted generalised additive model. The red line indicates those parts of the model fit that are statistically significantly changing and the shaded region shows the 95% confidence intervals.

To determine the lake surface areas at which a significant increase in the DTR occurs, a finite difference method was used to estimate the first derivatives of the fitted model (i.e. the model with only lake area as a predictor variable). This estimates the rate of change and, specifically, demonstrates when the rate of change is distinguishable from zero given the uncertainty in the model. The first derivative of the model identifies one clear region of statistically significant change in the DTR, which occurs at 3.2 x 10^6^ m^2^ (Fig 4). This period of significant change in the DTR was then superimposed on the observed data (e.g. Fig 3).

As well as exploring the influence of lake surface area, which was calculated to have the most significant effect on the DTR, we also tested other morphological characteristics of the lakes, such as volume and maximum depth, which are frequently covariant with lake surface area. However, these were all statistically less significant in describing variation in the DTR. For example, by using maximum depth instead of lake area, our statistical model explained only 73% of the variations in mean summer DTR; substantially less than the original model form. Due to the high correlation between lake volume and lake surface area (R^2^ = 0.96), substituting volume into Eq 3 did not alter the model output by much, although the deviance explained by the model was slightly lower. However, re-calculating the theoretical DTR (Eq 1) using lake volume instead of V_zmix_ demonstrated that the RMSE of the theoretical calculation increased from 0.6°C (i.e., using V_zmix_) to 1.53°C (i.e., using the entire lake volume), indicating that the DTR was more strongly related to the volume of the upper mixed layer than the lake volume.

Our dataset demonstrated that lake area exerted a significant influence on the DTR in lakes smaller than 3.2 x 10^6^ m^2^ (Figs 3 and 4). Small lakes vastly outnumber large lakes globally [33] and it has been estimated that 77% of lakes are of the order of 1 x 10^4^ m^2^ or smaller. Our results therefore emphasise that, in contrast to many of the world’s most studied lakes that tend to be large and thus strongly buffered thermally, the majority of lakes worldwide undergo marked diel variations. The DTR on individual days within the summer season can be significantly greater still. Fig 1C, for example, shows that the small Jekl Bog (surface area 2.5 x 10^3^ m^2^) had a DTR that can extend beyond 15°C on some days.

Given the importance of temperature in influencing biogeochemical processes and the distribution of many organisms [34], our findings highlight the likely importance of a lake’s dimensions in determining its ecological structure and function. Several studies have emphasised the role of lake size in shaping lake behaviour [35], including carbon cycling [8, 36] and species diversity [37], but the possibility that diel temperature variation, mediated by lake size, may be important in these differences and more generally has not previously been recognised. For example, large surface diel temperature cycles may have important implications for the assessment of temperature-dependent biogeochemical cycles influenced by lakes. In small lakes these processes need to be studied in a manner that accurately resolves the influence of temperature at diel timescales.

**Fig 5:**
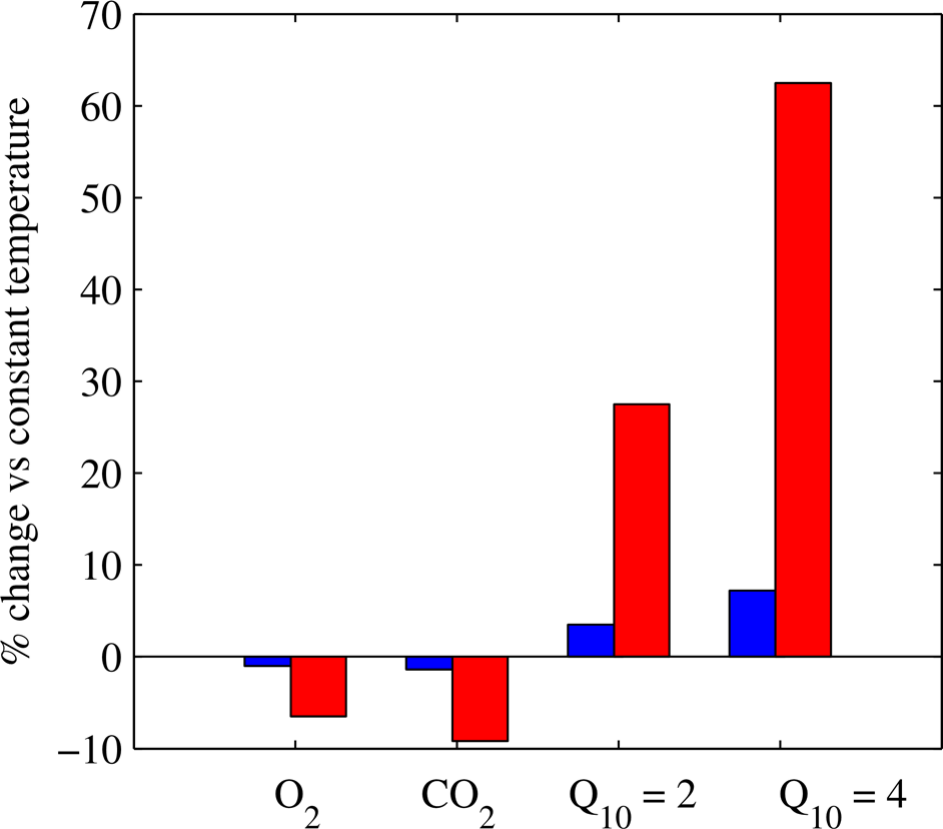
Estimated ecological and biogeochemical consequences of the diel surface temperature range. Potential bias in estimates of CO_2_ and O_2_ solubility and rates of processes with Q_10_ values of 2 or 4 for a diel temperature range of 1 (blue) or 7°C (red).

Many ecological and biochemical processes are non-linearly dependent on temperature, as illustrated by the ubiquitous use of Q_10_ values for representing rate changes. Literature values derived from analysis of global data sets suggest Q_10_ values of about 1.6 and 2.5 for photosynthesis and respiration respectively [5] and about 4 for methane emissions [10]. Where diel temperature cycles are large, but not resolved in data collection, errors in assessing the magnitude of processes may, therefore, also be large. If, for example, a DTR is only 1°C, as is typical of large lakes, estimation of daily means of solubility of O_2_ and CO_2_ and rates of processes with a Q_10_ of 2 or 4 from single point measurements could lead to errors of approximately 1% for O_2_ and CO_2_, and up to 4 and 7% for processes with a Q_10_ of 2 or 4, respectively (Fig 5). On the other hand, for small lakes, with diel cycles of, say, 7°C, errors could be up to 7 and 9% in the near-surface solubility of O_2_ and CO_2_, respectively, and up to 28 and 62% for the rate of processes with a Q_10_ of 2 and 4, respectively (Fig 5). On extreme days, such as those illustrated for Jekl Bog (Fig 1C) where diel temperature cycles can vary by up to 15°C, estimated rates for processes with a Q_10_ of 4 from a single measurement could be in error by over 180%. Not including diel variations in temperature within gas flux calculations could even lead to erroneous estimates of the direction of gas flux as well as the magnitude. In addition, single point measurements taken at variable times of day may not provide sufficiently representative values to make cross-lake comparisons or robust assessments of long-term trends in surface temperature (e.g. [38]). This is a key area of research that requires attention when conducting large-scale comparisons in lake temperature trends. This work, therefore, underlines the importance of conducting measurements at an appropriate scale in order to capture the full range of response of dynamic systems, such as small lakes, and highlights the need to increase research effort on these common but understudied systems.

We have shown, for the first time, dramatic differences between the diel upper water temperature cycles from a large distribution of lakes. While a number of factors can potentially influence the DTR, our observation that a single variable, surface area, exerts such a dominant influence on surface temperature variation will be of value in scaling up observations of impacts on temperature-dependent processes, and in elucidating systematic differences between large and small lakes. Light attenuation was also shown to influence the DTR, albeit much less strongly than to lake area. Light attenuation has, however, been shown not only to increase with decreasing lake size [39, 40], but also to have more effect on the depth of the epilimnion in smaller lakes than in larger ones [41], for which, except in the very clearest lakes, most of the solar radiation is likely to be absorbed in the mixed layer. Thus, even the influence of light attenuation on the DTR will be mediated by lake area. Increases in DOC have been experienced in many lakes over recent decades, probably as a result of recovery from acidification [42], and climate change is forecast to increase DOC concentration further [43]. Similarly, ongoing cultural eutrophication and remediation will alter phytoplankton concentrations in lakes and therefore also their light climate [20]. Accordingly, the DTR may change in the future as lakes become more or less transparent than today and mixing depths alter correspondingly. Similarly, as wind provides mixing energy for lakes and alters turbulent fluxes of heat then future changes that affect wind speed, such as development or deforestation (e.g. [44]), also have the potential to alter the DTR.

## Conclusions

The diel range in lake surface temperature has a potentially major bearing on lake biogeochemical and ecological processes but, until the recent proliferation of high-frequency temperature measurements, large-scale analysis of diel temperature variations was not possible. We show that in summer the average diel temperature range can reach 7°C in small lakes (15°C on individual days) and that the magnitude of the diel range decreases strongly with increasing lake area. This has the potential to be a major source of uncertainty for current estimates of important temperature-dependent ecological and biogeochemical processes in lakes.

## Supporting Information

S1 Fig Map showing the location of each lake (black filled circles) included in this investigation. (DOCX)

S1 Table General characteristics of the lakes studied in this investigation. Shown are the names of each lake, along with their calculated diel temperature range and mixed depth, their latitude (φ), surface area (A_0_), altitude above sea level (h), maximum depth (z_m_), and light attenuation coefficient (K_d_). Details of the measurements available for each lake are also provided.
(DOCX)

S2 Table Contact name for each lake included in this investigation. Contact information for any of the below listed data contributors can be obtained from R. Iestyn Woolway (riwoolway@gmail.com).
(DOCX)
