# Supplementary material for: Diel Surface Temperature Range Scales with Lake Size
Source: PLoS One. 2016 Mar 29;11(3):e0152466. doi: 10.1371/journal.pone.0152466 (PMC4811584; doi:10.1371/journal.pone.0152466)
Supplement: S1 Fig — (DOCX) [file pone.0152466.s001.docx]

**S1 Fig.** Map showing the location of each lake (black filled circles) included in this investigation.

**
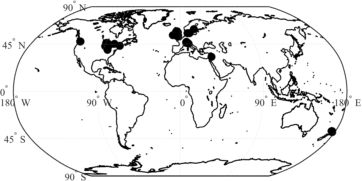
**
