# Supplementary material for: Diel Surface Temperature Range Scales with Lake Size
Source: PLoS One. 2016 Mar 29;11(3):e0152466. doi: 10.1371/journal.pone.0152466 (PMC4811584; doi:10.1371/journal.pone.0152466)
Supplement: S1 Table — Shown are the names of each lake, along with their calculated diel temperature range and mixed depth, their latitude (φ), surface area (A0), altitude above sea level (h), maximum depth (zm), and light attenuation coefficient (Kd). Details of the measurements available for each lake are also provided. (DOCX) [file pone.0152466.s002.docx]

**S1 Table. General characteristics of the lakes studied in this investigation.** Shown are the names of each lake, along with their calculated diel temperature range and mixed depth, their latitude (φ), surface area (A_0_), altitude above sea level (h), maximum depth (z_m_), and light attenuation coefficient (K_d_). Details of the measurements available for each lake are also provided.

| **Lake Name** | **Diel temperature range (°C)** | **Mixed depth (m)** | **φ (°N)** | **A_0_ (km^2^)** | **h (m)** | **z_m_ (m)** | **K_d_ (m^-1^)** | **Water temperature profile (°C)** | **Surface water temperature (°C)** | **Wind speed (m s^-1^)** | **Short-wave radiation (W m^-2^)** | **Relative humidity (%)** | **Air temperature (°C)** |
| --- | --- | --- | --- | --- | --- | --- | --- | --- | --- | --- | --- | --- | --- |
| Acton Lake | 1.58 |  | 39.5702 | 2.53 | 263 | 8.0 | 1.62 |  | ✓ |  |  |  |  |
| Bachsee | 1.56 |  | 46.67 | 0.0803 | 2265 | 18.8 | - |  | ✓ |  |  |  |  |
| Bänzlauiseeli, Unteres | 4.28 |  | 46.6897 | 0.0207 | 2177 | - | - |  | ✓ |  |  |  |  |
| Bassenthwaite Lake | 0.61 | 10.8 | 54.6537 | 5.3 | 69 | 19.0 | 0.55 | ✓ | ✓ | ✓ | ✓ | ✓ | ✓ |
| Blelham Tarn | 0.86 | 4.0 | 54.3961 | 0.1 | 45 | 15.0 | 0.64 | ✓ | ✓ | ✓ | ✓ | ✓ | ✓ |
| Bolger Lake | 3.56 |  | 46.2305 | 0.010633 | 483 | 3.5 | 5.1 |  | ✓ |  |  |  |  |
| Brotherswater | 1.09 |  | 54.5063 | 0.19 | 161 | 15.0 | 0.31 |  | ✓ |  |  |  |  |
| Burgäschisee | 2.26 |  | 47.1695 | 0.21 | 465 | 36.0 | - |  | ✓ |  |  |  |  |
| Burgseeli | 1.47 |  | 46.6975 | 0.0525 | 613 | 19.1 | - |  | ✓ |  |  |  |  |
| Burnmoor Tarn | 0.89 |  | 54.4285 | 0.239 | 252 | 13.0 | 0.49 |  | ✓ |  |  |  |  |
| Buttermere | 0.33 |  | 54.5312 | 0.93 | 100 | 23.0 | 0.16 |  | ✓ |  |  |  |  |
| Castle Lake | 1.29 | 4.4 | 55.934 | 0.223046 | 28 | 9.0 | 2.1 | ✓ | ✓ | ✓ | ✓ | ✓ | ✓ |
| Clatto Reservoir | 1.17 |  | 56.4991 | 0.094 | 153 | 7.0 | 0.65 |  | ✓ |  |  |  |  |
| Coniston Water | 0.62 |  | 54.3381 | 4.7 | 44 | 56.0 | 0.24 |  | ✓ |  |  |  |  |
| Crampton Lake | 0.99 | 5.3 | 46.2105 | 0.258925 | 512 | 18.5 | 0.81 | ✓ | ✓ | ✓ | ✓ | ✓ | ✓ |
| Crystal Bog | 4.83 | 1.1 | 46.0076 | 0.005524 | 503 | 2.25 | 3.45 |  | ✓ |  |  |  |  |
| Crystal Lake | 0.45 | 5.7 | 46.002 | 0.34 | 501 | 20.0 | 0.38 | ✓ | ✓ | ✓ | ✓ | ✓ | ✓ |
| Cwm Mynach | 1.64 |  | 52.7947 | 0.057 | 285 | 11.0 | 0.28 |  | ✓ |  |  |  |  |
| Dittligsee | 2.64 |  | 46.7564 | 0.0602 | 652 | 16.4 | - |  | ✓ |  |  |  |  |
| Egelmösli | 2.85 |  | 46.945 | 0.0152 | 550 | 3.4 | - |  | ✓ |  |  |  |  |
| Elterwater | 1.65 |  | 54.4272 | 0.17 | 53 | 7.0 | 0.69 |  | ✓ |  |  |  |  |
| Ennerdale Water | 0.54 |  | 54.5222 | 3 | 113 | 42.0 | 0.29 |  | ✓ |  |  |  |  |
| Esthwaite Water | 1.10 | 5.5 | 54.3583 | 1 | 66 | 16.0 | 0.56 | ✓ | ✓ | ✓ | ✓ | ✓ | ✓ |
| Flemington | 0.98 |  | 57.5424 | 0.14 | 40 | 2.35 | 4.8 |  | ✓ |  |  |  |  |
| Flueseeli | 3.52 |  | 46.4096 | 0.0347 | 2045 | 8.5 | - |  | ✓ |  |  |  |  |
| Gadenlauisee | 3.44 |  | 46.7209 | 0.0082 | 2155 | 7.5 | - |  | ✓ |  |  |  |  |
| Gantrischseeli | 3.56 |  | 46.7118 | 0.0141 | 1578 | 2.2 | - |  | ✓ |  |  |  |  |
| Gorm Lochan | 3.52 |  | 57.1719 | 0.0164 | 870 | - | - |  | ✓ |  |  |  |  |
| Grane Langsø | 1.37 |  | 56.0181 | 0.11 | 82 | 12.2 | 0.18 |  | ✓ |  |  |  |  |
| Grasmere | 1.09 |  | 54.4484 | 0.64 | 62 | 21.5 | 0.39 |  | ✓ |  |  |  |  |
| Hagelseeli | 4.29 |  | 46.6893 | 0.0064 | 2410 | - | - |  | ✓ |  |  |  |  |
| Hagelseewli | 1.78 |  | 46.673 | 0.0249 | 2339 | 18.8 | - |  | ✓ |  |  |  |  |
| Harp Lake | 1.10 |  | 45.3796 | 1.513724 | 327 | 19.0 | 0.64 |  | ✓ |  |  |  |  |
| Haxeseeli | 2.39 |  | 46.6803 | 0.0243 | 2464 | - | - |  | ✓ |  |  |  |  |
| Hinterburgseeli | 2.35 |  | 46.718 | 0.045 | 1514 | 11.4 | - |  | ✓ |  |  |  |  |
| Hummingbird | 4.18 |  | 46.2437 | 0.007862 | 516 | 7.6 | 5.7 |  | ✓ |  |  |  |  |
| Jekl Bog | 8.34 |  | 45.9945 | 0.002468 | 499 | 2.5 | 1.9 |  | ✓ |  |  |  |  |
| Lake Erken | 0.30 | 11.6 | 59.846 | 23.6742 | 11 | 20.7 | 0.4 | ✓ | ✓ | ✓ | ✓ | ✓ | ✓ |
| Lake Iseo | 0.94 | 8.0 | 45.724 | 60.9 | 185 | 256 | 0.49 | ✓ | ✓ | ✓ | ✓ | ✓ | ✓ |
| Lake Kinneret | 0.65 | 14.1 | 32.817 | 164.25 | -211 | 43 | 0.5 | ✓ | ✓ | ✓ | ✓ | ✓ | ✓ |
| Lake Mendota | 0.78 | 6.9 | 43.105 | 38.2824 | 259 | 25.0 | 0.82 | ✓ | ✓ | ✓ | ✓ | ✓ | ✓ |
| Lake Ngaroto | 0.88 |  | -37.954 | 1.08 | 33 | 4.0 | 3.6 |  | ✓ |  |  |  |  |
| Lake Rotoiti | 0.67 | 8.9 | -38.037 | 34.6 | 279 | 94.0 | 0.54 | ✓ | ✓ | ✓ | ✓ | ✓ | ✓ |
| Lake Rotorua | 0.37 | 15.5 | -38.082 | 79.8555 | 280 | 22.0 | 0.63 | ✓ | ✓ | ✓ | ✓ | ✓ | ✓ |
| Lake Sunapee | 0.69 | 7.5 | 43.391 | 16.2489 | 333 | 33.0 | 0.39 | ✓ | ✓ | ✓ | ✓ | ✓ | ✓ |
| Lake Tarawera | 0.59 |  | -38.198 | 41 | 298 | 88.0 | 0.08 |  | ✓ |  |  |  |  |
| Lake Union | 0.49 |  | 47.639 | 2.35 | 5 | 15.0 | 0.33 |  | ✓ |  |  |  |  |
| Lake Waikaremoana | 0.44 | 19.1 | -38.768 | 54 | 600 | 256.0 | 0.13 | ✓ | ✓ | ✓ | ✓ | ✓ | ✓ |
| Lake Washington | 0.66 |  | 47.618 | 88 | 0 | 65.0 | 0.2 |  | ✓ |  |  |  |  |
| Lake Wingra | 1.13 |  | 43.0537 | 1.30 | 257 | 4.3 | 3.2 |  | ✓ |  |  |  |  |
| Lake Wintergreen | 1.83 | 2.7 | 42.398 | 0.164 | 275 | 7.9 | 1.3 | ✓ | ✓ | ✓ | ✓ | ✓ | ✓ |
| Lawrence Lake | 1.76 | 3.3 | 42.4409 | 0.051 | 274 | 12.3 | 0.53 | ✓ | ✓ | ✓ | ✓ | ✓ | ✓ |
| Llyn Celyn | 0.55 |  | 52.9493 | 3.236 | 299 | 43.0 | - |  | ✓ |  |  |  |  |
| Llyn Conwy | 0.48 |  | 52.9993 | 0.4 | 454 | 19.0 | 0.54 |  | ✓ |  |  |  |  |
| Llyn Llagi | 1.64 |  | 53.0145 | 0.051 | 380 | 15.0 | 0.34 |  | ✓ |  |  |  |  |
| Llyn Padarn | 0.89 |  | 53.128 | 0.97 | 316 | 29.0 | 0.48 |  | ✓ |  |  |  |  |
| Llyn Tegid | 0.93 |  | 52.8885 | 4.1 | 164 | 43.0 | 0.58 |  | ✓ |  |  |  |  |
| Loch a’ Mhadaidh | 1.37 |  | 57.7126 | 0.311 | 570 | 46.0 | 0.25 |  | ✓ |  |  |  |  |
| Loch an Fhuar-thill Mhoir | 3.70 |  | 57.4524 | 0.0536 | 770 | 15.0 | 0.36 |  | ✓ |  |  |  |  |
| Loch Bhuic Moir | 1.54 |  | 57.2785 | 0.0355 | 540 | 7.8 | 0.6 |  | ✓ |  |  |  |  |
| Loch Chon | 0.56 |  | 56.213 | 1.057 | 96 | 25.0 | 0.55 |  | ✓ |  |  |  |  |
| Loch Coire Fionnaraich | 0.84 |  | 57.4921 | 0.093 | 236 | 14.0 | 0.55 |  | ✓ |  |  |  |  |
| Loch Coire Mhic Fherchair | 0.84 |  | 57.5912 | 0.1005 | 600 | 24.0 | 0.13 |  | ✓ |  |  |  |  |
| Loch Gorm | 2.23 |  | 57.6813 | 0.2161 | 540 | 47.0 | 0.25 |  | ✓ |  |  |  |  |
| Loch Grannoch | 0.89 |  | 55.0021 | 1.114 | 214 | 21.0 | 0.8 |  | ✓ |  |  |  |  |
| Loch Lomond | 0.38 |  | 56.105 | 71 | 7.6 | 189.0 | 0.53 |  | ✓ |  |  |  |  |
| Loch Nagar | 0.51 |  | 56.959 | 0.099 | 788 | 27.0 | 0.19 |  | ✓ |  |  |  |  |
| Loch nan Eun | 1.08 |  | 56.885 | 0.1446 | 790 | 10.0 | 0.85 |  | ✓ |  |  |  |  |
| Loch Tinker | 1.07 |  | 56.2273 | 0.11 | 418 | 10.0 | 0.43 |  | ✓ |  |  |  |  |
| Loch Toll Lochan | 2.07 |  | 57.4955 | 0.0755 | 520 | 14.0 | 0.72 |  | ✓ |  |  |  |  |
| Lochan a’ Chnapaich | 1.20 |  | 57.8098 | 0.056 | 690 | 8.8 | 0.39 |  | ✓ |  |  |  |  |
| Lough Feeagh | 0.47 | 10.8 | 53.943 | 3.327559 | 14 | 45.0 | 0.96 | ✓ | ✓ | ✓ | ✓ | ✓ | ✓ |
| Loweswater | 0.70 | 9.1 | 54.5825 | 0.6 | 121 | 16.0 | 0.5 | ✓ | ✓ | ✓ | ✓ | ✓ | ✓ |
| Meienfallseeli | 4.08 |  | 46.5869 | 0.0152 | 1900 | - | - |  | ✓ |  |  |  |  |
| Moossee | 2.25 |  | 47.0221 | 0.3035 | 521 | 21.1 | - |  | ✓ |  |  |  |  |
| Morris Lake | 2.71 |  | 46.2575 | 0.058819 | 506 | 6.7 | 2.63 |  | ✓ |  |  |  |  |
| Mouser Bog | 3.51 | 1.2 | 45.998 | 0.037833 | 494 | 4.3 | 2.44 | ✓ | ✓ | ✓ | ✓ | ✓ | ✓ |
| North Sparkling Bog | 6.47 |  | 46.005 | 0.005 | 497 | 4.3 | 2.54 |  |  |  |  |  |  |
| Oberstockensee | 1.49 |  | 46.6872 | 0.1181 | 1665 | 43.0 | - |  | ✓ |  |  |  |  |
| Priest Pot | 5.63 |  | 54.3721 | 0.01 | 66 | 3.5 | 1.44 |  | ✓ |  |  |  |  |
| Rostherne Mere | 0.61 |  | 53.354 | 0.5 | 27 | 30.0 | 0.85 |  | ✓ |  |  |  |  |
| Round Loch of Glenhead | 1.27 |  | 55.0933 | 0.127 | 298 | 14.0 | 0.39 |  | ✓ |  |  |  |  |
| Sägistalsee | 1.85 |  | 46.6796 | 0.0725 | 1935 | 9.4 | - |  | ✓ |  |  |  |  |
| Sammamish | 0.45 |  | 47.6 | 19.8 | 9 | 32.0 | 0.34 |  | ✓ |  |  |  |  |
| Schwarzsee | 1.60 |  | 46.6657 | 0.4554 | 1046 | 10.0 | - |  | ✓ |  |  |  |  |
| Scoat Tarn | 1.08 |  | 54.4812 | 0.043 | 602 | 20.0 | 0.17 |  | ✓ |  |  |  |  |
| Seebergsee | 2.33 |  | 46.5774 | 0.0576 | 1831 | 15.3 | - |  | ✓ |  |  |  |  |
| Seebodensee | 3.71 |  | 46.7235 | 0.0073 | 2042 | 3.0 | - |  | ✓ |  |  |  |  |
| South Trout Lake | 0.47 | 8.3 | 46.029 | 14.9184 | 495 | 34.0 | 0.39 | ✓ | ✓ | ✓ | ✓ | ✓ | ✓ |
| Sparkling Lake | 0.80 | 6.5 | 46.008 | 0.618453 | 497 | 20.0 | 0.39 | ✓ | ✓ | ✓ | ✓ | ✓ | ✓ |
| St. Gribsø | 1.50 | 2.5 | 55.985 | 0.100285 | 50 | 12.0 | 2.3 | ✓ | ✓ | ✓ | ✓ | ✓ | ✓ |
| Strathclyde Loch | 1.22 |  | 55.793 | 0.82 | 18 | 7.0 | - |  | ✓ |  |  |  |  |
| Sulsseewli | 5.40 |  | 46.6175 | 0.0201 | 1920 | - | - |  | ✓ |  |  |  |  |
| Sulsseewli, Oberes | 4.58 |  | 46.6121 | 0.0072 | 2191 | 2.4 | - |  | ✓ |  |  |  |  |
| Triebtenseewli | 1.66 |  | 46.5527 | 0.0966 | 2365 | 24.0 | - |  | ✓ |  |  |  |  |
| Trout Bog | 6.62 | 1.1 | 46.041 | 0.010478 | 495 | 8.0 | 2.89 | ✓ | ✓ | ✓ | ✓ | ✓ | ✓ |
| Ullswater | 0.86 |  | 54.5761 | 8.90 | 145 | 63.0 | 0.32 |  | ✓ |  |  |  |  |
| unnamed (SC0084) | 2.23 |  | 57.5168 | 0.0964 | 670 | 32.0 | 0.16 |  | ✓ |  |  |  |  |
| West Long Lake | 2.43 |  | 46.2356 | 0.048658 | 513 | 14.0 | 1.58 |  | ✓ |  |  |  |  |
| Windermere South Basin | 0.56 | 11.5 | 54.35 | 6.7 | 39 | 42.0 | 0.45 | ✓ | ✓ | ✓ | ✓ | ✓ | ✓ |
