# Supplementary material for: Diel Surface Temperature Range Scales with Lake Size
Source: PLoS One. 2016 Mar 29;11(3):e0152466. doi: 10.1371/journal.pone.0152466 (PMC4811584; doi:10.1371/journal.pone.0152466)
Supplement: S2 Table — Contact information for any of the below listed data contributors can be obtained from R. Iestyn Woolway (riwoolway@gmail.com). (DOCX) [file pone.0152466.s003.docx]

**S2 Table.** Contact name for each lake included in this investigation. Contact information for any of the below listed data contributors can be obtained from R. Iestyn Woolway (riwoolway@gmail.com).

| Lake | Contact |
| --- | --- |
| Acton Lake | Kevin Rose |
| Bachsee | David Livingstone |
| Banzlauiseeli, Unteres | David Livingstone |
| Bassenthwaite Lake | Stephen Maberly |
| Blelham Tarn | Stephen Maberly |
| Bolger Lake | Jake Zwart |
| Brotherswater | Stephen Maberly |
| Burgaschisee | David Livingstone |
| Burgseeli | David Livingstone |
| Burnmoor Tarn | Ewan Shilland |
| Buttermere | Stephen Maberly |
| Castle Lake | Peter Staehr |
| Clatto Reservoir | Stephen Maberly |
| Coniston Water | Stephen Maberly |
| Crampton Lake | Jake Zwart |
| Crystal Bog | NTL-LTER |
| Crystal Lake | NTL-LTER |
| Cwm Mynach | Ewan Shilland |
| Dittligsee | David Livingstone |
| Egelmosli | David Livingstone |
| Elterwater | Stephen Maberly |
| Ennerdale Water | Stephen Maberly |
| Esthwaite Water | Stephen Maberly |
| Flemington | Stephen Maberly |
| Flueseeli | David Livingstone |
| Gadenlauisee | David Livingstone |
| Gantrischseeli | David Livingstone |
| Gorm Lochan | Ewan Shilland |
| Grane Langso | Mikkel Andersen |
| Grasmere | Stephen Maberly |
| Hagelseeli | David Livingstone |
| Hagelseewli | David Livingstone |
| Harp Lake | James Rusak |
| Haxeseeli | David Livingstone |
| Hinterburgseeli | David Livingstone |
| Hummingbird | Jake Zwart |
| Jekl Bog | NTL-LTER |
| Lake Erken | Gesa Weyhenmeyer |
| Lake Iseo | http://hydraulics.unibs.it/hydraulics/?amp%3b2269 |
| Lake Kinneret | Alon Rimmer |
| Lake Mendota | NTL-LTER |
| Lake Ngaroto | David Hamilton |
| Lake Rotoiti | David Hamilton |
| Lake Rotorua | David Hamilton |
| Lake Sunapee | Kathie Weathers |
| Lake Tarawera | David Hamilton |
| Lake Union | Curtis DeGasperi |
| Lake Waikaremoana | David Hamilton |
| Lake Washington | Curtis DeGasperi |
| Lake Wingra | NTL-LTER |
| Lake Wintergreen | Robyn Smyth |
| Lawrence Lake | Robyn Smyth |
| Llyn Celyn | Ian Williams |
| Llyn Conwy | Chris Evans |
| Llyn Llagi | Ewan Shilland |
| Llyn Padarn | Tanya Kitteridge |
| Llyn Tegid | Rhian Thomas |
| Loch a' Mhadaidh | Martin Kernan |
| Loch an Fhuar-thill Mhoir | Martin Kernan |
| Loch Bhuic Moir | Martin Kernan |
| Loch Chon | Ewan Shilland |
| Loch Coire Fionnaraich | Ewan Shilland |
| Loch Coire Mhic Fherchair | Martin Kernan |
| Loch Gorm | Ewan Shilland |
| Loch Grannoch | Ewan Shilland |
| Loch Lomond | Susan Waldron |
| Loch Nagar | Ewan Shilland |
| Loch nan Eun | Martin Kernan |
| Loch Tinker | Ewan Shilland |
| Loch Toll Lochan | Martin Kernan |
| Lochan a' Chnapaich | Martin Kernan |
| Lough Feeagh | Elvira de Eyto |
| Loweswater | Stephen Maberly |
| Meienfallseeli | David Livingstone |
| Moossee | David Livingstone |
| Morris Lake | Jake Zwart |
| Mouser Bog | NTL-LTER |
| North Sparkling Bog | NTL-LTER |
| Oberstockensee | David Livingstone |
| Priest Pot | Stephen Maberly |
| Rostherne Mere | Dave Ryves |
| Round Loch of Glenhead | Ewan Shilland |
| Sagistalsee | David Livingstone |
| Sammamish | Curtis DeGasperi |
| Schwarzsee | David Livingstone |
| Scoat Tarn | Ewan Shilland |
| Seebergsee | David Livingstone |
| Seebodensee | David Livingstone |
| South Trout Lake | NTL-LTER |
| Sparkling Lake | NTL-LTER |
| St. Gribso | Peter Staehr |
| Strathclyde Loch | Jan Krokowski |
| Sulsseewli | David Livingstone |
| Sulsseewli, Oberes | David Livingstone |
| Triebtenseewli | David Livingstone |
| Trout Bog | NTL-LTER |
| Ullswater | Stephen Maberly |
| unnamed (SC0084) | Martin Kernan |
| West Long Lake | Jake Zwart |
| Windermere South Basin | Stephen Maberly |
